# Quantitative detection of hydrogen peroxide in rain, air, exhaled breath, and biological fluids by NMR spectroscopy

**DOI:** 10.1073/pnas.2121542119

**Published:** 2022-02-14

**Authors:** Tayeb Kakeshpour, Belhu Metaferia, Richard N. Zare, Adriaan Bax

**Affiliations:** ^a^Laboratory of Chemical Physics, National Institute of Diabetes and Digestive and Kidney Diseases, NIH, Bethesda, MD 20892;; ^b^Department of Chemistry, Stanford University, Stanford, CA 94305

**Keywords:** hydrogen peroxide, microdroplet, exhaled breath condensate, NMR spectroscopy, respiratory droplet

## Abstract

Hydrogen peroxide (H_2_O_2_) plays a key role in environmental chemistry, biology, and medicine. H_2_O_2_ concentrations typically are 6 to 10 orders of magnitude lower than that of water, making its quantitative detection challenging. We demonstrate that optimized NMR spectroscopy allows direct, interference-free, quantitative measurements of H_2_O_2_ down to submicromolar levels in a wide range of fluids, ranging from exhaled breath and air condensate to rain, blood, urine, and saliva. NMR measurements confirm the previously reported spontaneous generation of H_2_O_2_ in microdroplets that form when condensing water vapor on a hydrophobic surface, which can interfere with atmospheric H_2_O_2_ measurements. Its antimicrobial activity and strong seasonal variation speculatively could be linked to the seasonality of respiratory viral diseases.

Hydrogen peroxide (H_2_O_2_) is a vital chemical oxidant that plays key roles in many aspects of life, ranging from environmental chemistry to human health. Levels in blood hold diagnostic potential for thyroid disease (1) and have been linked to other maladies, including Alzheimer’s disease (2) and cancer (3). Up-regulation of H_2_O_2_ production by neutrophils and macrophages in the respiratory epithelium in response to inhaled pathogens mediates inflammation by activating nuclear factor kappa-light-chain-enhancer of activated B cells (NF*κ*B), a key regulator of the innate immune system (4). Intriguingly, the enzyme myeloperoxidase, whose production is also up-regulated by neutrophils upon infection, converts H_2_O_2_ in the presence of chloride into hypochlorite, which may act directly on the pathogen but also has been implicated in the pathogenesis associated with the acute respiratory distress syndrome (5). Elevated levels of H_2_O_2_ in exhaled breath condensate (EBC) have been linked to asthma and chronic obstructive pulmonary disease (6). However, detection of H_2_O_2_ in breath can be impacted by H_2_O_2_’s variable presence in the atmosphere (7).

Quantitative detection of trace amounts of H_2_O_2_ commonly relies on the formation of fluorescent products (8), a process that can suffer from chemical interference, such as quenching, incomplete reactions, or reaction with other reactive oxygen species, which may have contributed to large discrepancies in previously reported values. For example, H_2_O_2_ concentrations in blood range anywhere from 0.2 nM to 100 mM (9), and values reported for rain (0.03 µM to 199 µM) and ground-level air (0.02 parts per billion by volume [ppbv] to 180 ppbv) also cover nearly four orders of magnitude (7). The recent discovery of spontaneous generation of levels as high as 100 µM H_2_O_2_ upon condensation of water vapor into microdroplets (10) also may interfere with quantitation. Indeed, the very wide range of H_2_O_2_ concentrations reported for EBC may also include generation of H_2_O_2_ in microdroplets formed in the collection device.

We employ NMR spectroscopy to readily quantify H_2_O_2_ down to submicromolar concentrations. NMR has been used previously for H_2_O_2_ quantification (11–13), but not for detecting trace amounts. Here, we report its application to samples of interest in medicine and atmospheric chemistry. By taking advantage of fast hydrogen exchange (HX) of H_2_O_2_ hydrogens with those of water, the speed of NMR data acquisition can be increased to *ca*. 1,000 scans per minute (14). When combined with improvements in NMR hardware, we achieve a strong enhancement in the sensitivity of H_2_O_2_ detection, enabling quantitative measurement in biological fluids.

## Results and Discussion

First, we demonstrate NMR measurement of H_2_O_2_ in EBC and how microdroplet formation on the hydrophobic surface of the collection device contributes to the measured concentration. We used a commercial R-tube EBC collection device (https://respiratoryresearch.com/rtube/) where water vapor condenses on the inside of a cooled plastic tube. At the start of the condensation process, microdroplets form on the hydrophobic, initially dry inner surface of the R-tube cylinder. Analogous formation of microdroplets on a hydrophobic surface yields H_2_O_2_ concentrations in water as high as 100 µM (10), considerably higher than values expected for EBC. However, the initial total volume of microdroplets associated with H_2_O_2_ generation, V_1_, is small, and, once droplets become larger, spontaneous H_2_O_2_ generation decreases to zero (10). Therefore, subsequent condensation dilutes this initial burst of H_2_O_2_ with water vapor that contains true EBC levels of H_2_O_2_.

**Fig. 1.**
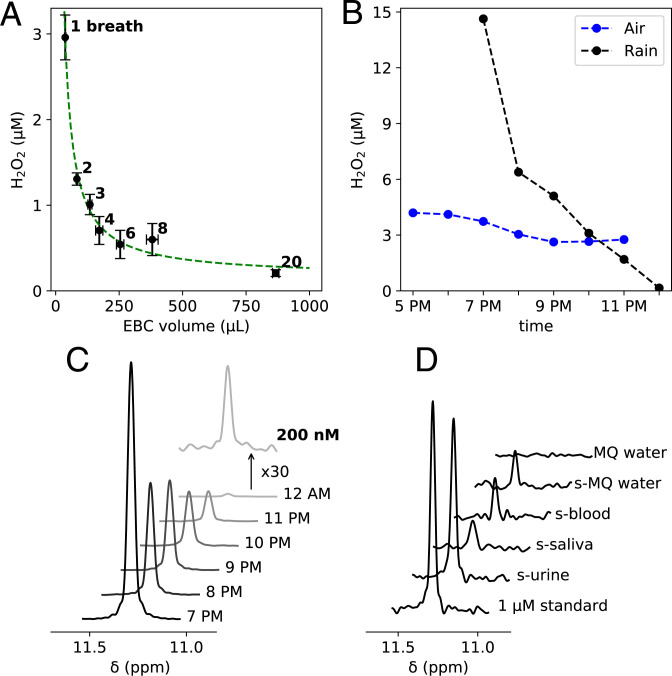
NMR detection of H_2_O_2_ in various fluids. (*A*) Dependence of H_2_O_2_ concentration in EBC on the collected volume. The green dashed line represents the best fit to Eq. **1**. (*B*) H_2_O_2_ levels in rainwater and samples condensed from ambient air on April 24, 2021. (*C*) NMR spectra of the rainwater samples. (*D*) NMR spectra of a 1-µM standard sample, sublimed (s-)urine, s-saliva, s-blood, s-MQ water, and MQ water.

Fig. 1*A* displays this behavior: The H_2_O_2_ concentration in EBC rapidly decreases to <1 µM with increasing numbers of breaths and total collected condensate. Under these conditions, the averaged concentration, [H_2_O_2_], can be written as

[1] $$[{\text{H}}_{2}{\text{O}}_{2}]=({\text{C}}_{1}{\text{V}}_{1}+{\text{C}}_{2}{\text{V}}_{2})/({\text{V}}_{1}+{\text{V}}_{2})\approx {\text{N}}_{1}/{\text{V}}_{2}+{\text{C}}_{2},$$

in which C and V denote H_2_O_2_ concentration (moles per liter) and condensate volume (liter); subscripts 1 and 2 refer to the microdroplets initially formed on the surface of the R tube, and subsequently formed by additional vapor condensation; and N is the amount (moles). Because V_2_ is much larger than V_1_, and the minimum amount of liquid that can reproducibly be harvested from the device is *ca*. 25 µL, independent determination of C_1_ and V_1_ is imprecise. Only the product C_1_V_1_ = N_1_ and the EBC H_2_O_2_ concentration can be reliably extracted from the data, yielding values of N_1_ = 104 ± 5 pmol and C_2_ = 0.16 ± 0.06 µM when H_2_O_2_-free dry air (relative humidity [RH] < 1%) was inhaled. The surface area of the inside of the R tube is 123 cm^2^, and a rough estimate for the aqueous volume of the microdroplets, V_1_, formed on the inside of the R tube considers their total volume equivalent to that of a homogeneous water layer of 1-µm thickness, yielding C_1_ = 104 pmol/12.3 µL = 8.5 µM. This value is in fair agreement with exhaling a single shallow breath into a falcon tube (polypropylene) followed by centrifuging the condensation fog to the bottom (repeated 20 times), which yielded 12.7 µM H_2_O_2_. The H_2_O_2_ level of EBC may also depend on its concentration in the inhaled air source (15). The concentration of H_2_O_2_ in the condensate from 20 breaths using H_2_O_2_-free dry air was 0.21 ± 0.04 µM, slightly smaller than the 0.27 ± 0.05 µM value obtained using ambient air (RH 25%) that contained 4 µM H_2_O_2_ in its condensate.

The same R-tube device was used for quantifying H_2_O_2_ in ambient air. We slightly modified the commercial R-tube device (details in *Materials and Methods*), which allowed us to pass a constant airflow through it while the aluminum cooling sleeve was in contact with dry ice. On April 21, 2021, we started collecting indoor air samples about 2 h before the forecasted rain. From 5 PM to 6 PM (Fig. 1*B*), the air H_2_O_2_ level was constant at around 4.2 µM. However, as soon as rain started around 6 PM, it decreased to 2.6 µM over the course of 2 h. We were able to collect the first rain sample at 7 PM briefly after the rain started. This sample contained 14.6 µM of H_2_O_2_, but it decreased to as low as 0.2 µM at its endpoint (Fig. 1 *B* and *C*). Such high levels of H_2_O_2_ in the initial stages of rain and its subsequent decline agree with prior observations (16) and mirror a rain sample collected at the start of Hurricane Ida (September 1, 2021; Bethesda, MD) that contained 56.5 µM H_2_O_2_.

Arguably, blood represents the most important but also the most challenging fluid for quantitative measurement of [H_2_O_2_]. Its relatively high ionic strength, including HX-catalyzing phosphate ions that hinder NMR observation, and chemical substances, including the enzymes catalase and glutathione peroxidase that regulate [H_2_O_2_], can interfere with the measurement. In addition, conversion of superoxide into H_2_O_2_ by superoxide dismutase can increase [H_2_O_2_]. To avoid disturbing the equilibrium [H_2_O_2_] in blood, we fast froze a freshly collected ( <3 min) human blood sample into liquid isopentane at *ca*. –140 ^∘^C. The water and H_2_O_2_ were then sublimed from the blood while keeping the blood frozen, thereby also removing molecules with functional groups that promote HX (*Materials and Methods*). Human whole blood contains 77 to 82% water by weight, and we sublimed 78% of the whole blood mass. The deposited water on the cold finger contained 0.19 µM H_2_O_2_ (Fig. 1*D*), considerably lower than most literature values (9). However, sublimation of the same volume of milli-Q (MQ) water under identical conditions yields a very similar H_2_O_2_ concentration (Fig. 1*D* and Table 1), indicating that, analogous to vapor condensation (10), sublimation also generates H_2_O_2_. Therefore, the true blood H_2_O_2_ level must have been considerably below its measured value. Similarly, a sublimed sample of saliva contained about the same amount of H_2_O_2_, suggesting an upper limit of ≤ 100 nM. Sublimation of a urine sample resulted in 0.77 µM H_2_O_2_, suggesting at least 0.58 µM H_2_O_2_ in urine itself (Fig. 1*D* and Table 1).

**Table 1.** Observed H_2_O_2_ concentrations in various fluids

Substance	[H_2_O_2_] (µM)
EBC (20 breaths, dry air)*	0.21 ± 0.04
EBC (20 breaths, ambient air)*^,^^†^	0.27 ± 0.05
Rain	0.2–56.5
Ambient air	2.6–4.2
MQ water	≤ 0.02
Sublimed MQ water^‡^	0.19 ± 0.02
Sublimed saliva	≤ 0.2
Sublimed blood	≤ 0.2
Sublimed urine^§^	0.38–0.77

* Uncertainties obtained from triplicate measurements.

^†^[H_2_O_2_] remained *ca*. 4 µM during EBC collection.

^‡^Uncertainty is calculated from signal to noise.

^§^Values are not corrected for H_2_O_2_ generation during sublimation.

Our measurements demonstrate that NMR spectroscopy is a quite sensitive method for quantitative detection of [H_2_O_2_] in fluids. Because the method directly observes the H_2_O_2_ hydrogen signal that falls in a remote spectral region, it does not suffer from chemical interference or resonance overlap. We anticipate that quantitation of H_2_O_2_ by NMR spectroscopy will prove valuable not only for direct measurement but also as a “gold standard” to calibrate other methods that have resulted in highly divergent sets of literature [H_2_O_2_] values.

Prior to their desiccation, atmospheric H_2_O_2_ is in rapid exchange with the aqueous fraction of airborne droplets. Respiratory fluids contain myeloperoxidase, which converts H_2_O_2_ into the much more powerful hypochlorite oxidant, as well as catalase, which breaks down H_2_O_2_. Continuous influx of atmospheric H_2_O_2_ possibly could result in antimicrobial levels of hypochlorite, but only at RH levels that prevent evaporation for a sufficient time for reaction to cause pathogen inactivation (17) in droplets. Although elevated levels of H_2_O_2_ are known to be an airway irritant, the naturally occurring summertime H_2_O_2_ levels may play a role in limiting the duration airborne virus remains viable when humidity is sufficiently high to prevent rapid desiccation of respiratory droplets.

## Materials and Methods

### NMR Spectroscopy.

NMR samples contained 1 mM 2-(*N*-morpholino) ethanesulfonic acid (MES) buffer and 2% vol/vol D_2_O, pH 6.00 ± 0.05 (14). A low concentration of MES buffer was used to reduce catalysis of HX. Spectra were recorded at 2 ^∘^C to further minimize HX and retain a fairly narrow H_2_O_2_ resonance (line width *ca*. 15 Hz to 20 Hz). Selective excitation of the downfield spectral region, using a 3-ms Gaussian pulse, prevented excitation of the H_2_O resonance. Even under conditions that minimize HX of H_2_O_2_ with water, HX remains sufficiently fast to allow rapid signal averaging without saturating the H_2_O_2_ resonance (14). Spectra were recorded with 30,720 scans in *ca*. 30 min each, using a 700-MHz Bruker Avance-III NMR spectrometer equipped with a cryogenic probehead.

### Air Condensate and Rainwater Collection.

A commercial R tube was modified slightly for air sample collection. The mouthpiece was replaced by a septum connecting a hose to the input of the R tube to enable controlling the ambient airflow rate through the R tube using a gauge. Another septum was used at the exit of the R tube, allowing pulling of air through the device using a house vacuum line. An airflow of 10 L/min was passed through the R tube for 30 min while inserted into the aluminum sleeve in contact with dry ice. For rainwater collection, the narrow end of a plastic funnel was fitted into a 15-mL falcon tube.

### EBC Collection.

A K-type thermocouple was attached to the outside of the cooling aluminum sleeve. The sleeve was first cooled to –5 ^∘^C in a freezer, followed by slowly warming to 0 ^∘^C. During this time, eight deep inhalations of dry ( <1% RH), high-efficiency-particulate-air (HEPA)–filtered air were made, followed by nose exhalation. As soon as the cooling sleeve reached 0 ^∘^C, the mouthpiece, which was slightly warmed by a heat gun to prevent condensation, was connected to the R tube, the R tube was inserted into the sleeve, and breathing started with an inhale through the mouthpiece of the device which is equipped with its own unidirectionally valved HEPA filter. During sample collection, each deep inhalation (*ca*. 4 L) was timed to take 5 s, followed by 3 s of breath holding, and exhaling at an approximately constant flow for 12 s. The total time of each breath cycle was 20 s. For quantifying H_2_O_2_ formation on a polypropylene surface, single exhales were performed into four 50-mL falcon tubes at room temperature, followed by centrifugation (20,000 × *g*, 20 ^∘^C). This procedure was repeated five times to yield a total of 110 µL of EBC (i.e., 5.5 µL per exhale). EBC samples smaller than 500 µL were supplemented with MQ water to permit measurement in standard NMR sample tubes.

### Blood, Urine, and Saliva Sample Preparation.

Within 3 min of drawing, fresh human blood (*ca*. 1 mL) was added dropwise to magnetically stirred isopentane (20 mL) cooled to *ca*. –140 ^∘^C in a 100-mL flask. The addition was performed under an inert atmosphere using an N_2_ balloon. The flask was then stored at dry ice temperature before it was opened in a N_2_-filled glove bag for removing isopentane from the solid blood pellets, using a pipette. Next, a sublimation cold finger equipped with a vacuum valve was inserted into the flask (Sigma-Aldrich; product numbers Z129607 and Z515574), and the remaining isopentane was removed using a lyophilizer while keeping the bottom of the flask in contact with dry ice. Subsequently, the vacuum valve was closed, and the sample was sublimed by warming the frozen blood to about –15 ^∘^C to –10 ^∘^C and running liquid N_2_ through the cold finger for 4 h. Then, the sublimation device was filled with N_2_ gas and warmed to ambient temperature. During this time, the deposited ice on the cold finger thawed and was collected in a cup at the tip of the cold finger (875 mg). The fraction of the blood that did not sublime (245 mg) remained fully dehydrated after warming to ambient temperature, indicating completeness of the sublimation process. Urine and saliva samples were processed analogously to blood, and liquid fractions of 95 and 98 wt/wt% were collected on the cold finger after sublimation, consistent with their respective known water contents. All samples were deidentified.

## Data Availability

All study data are included in the main text.
